# Supplementary figures and images for: Repertoire of morphable proteins in an organism
Source: PeerJ. 2020 Feb 11;8:e8606. doi: 10.7717/peerj.8606 (PMC7020816; doi:10.7717/peerj.8606)

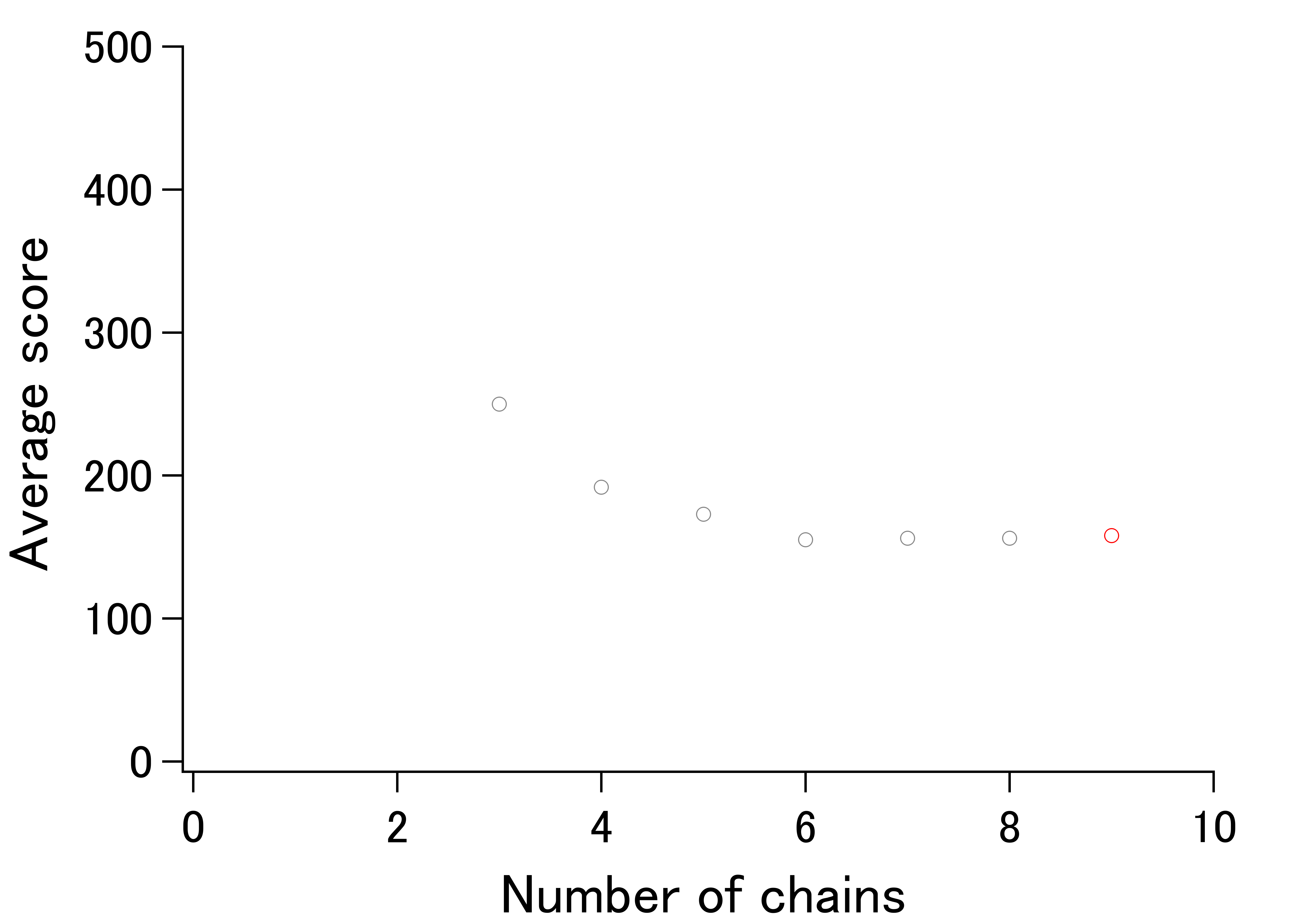

Supplement: Supplemental Information 4 — UMF for AAT of E. coli is the average score calculated using nine chains (red). [file peerj-08-8606-s004.png]
